# Optical monitoring of cerebral microcirculation in neurointensive care

**DOI:** 10.1007/s11517-017-1725-8

**Published:** 2017-12-08

**Authors:** Peter Rejmstad, Neda Haj-Hosseini, Oscar Åneman, Karin Wårdell

**Affiliations:** 1 Department of Biomedical Engineering, Linköping University, Linköping, Sweden; 2 Department of Neurosurgery, Linköping University Hospital, Linköping, Sweden; 3 Department of Clinical and Experimental Medicine, Linköping University, Linköping, Sweden

**Keywords:** Neurointensive care, Cerebral blood flow (CBF), Oxygenation, Diffuse reflectance spectroscopy (DRS), Laser Doppler flowmetry (LDF), Subarachnoid hemorrhage (SAH)

## Abstract

Continuous optical monitoring of local cerebral microcirculation could benefit neurointensive care patients treated for subarachnoid hemorrhage (SAH). The aim of the study was to evaluate laser Doppler flowmetry (LDF) and diffuse reflectance spectroscopy (DRS) for long-term monitoring of brain microcirculation and oxygen saturation (SO_2_) in the neurointensive care unit (NICU). A fiber optic probe was designed for intraparenchymal use and connected to LDF and DRS for assessment of the local blood flow (perfusion and tissue reflectance (TLI)) and SO_2_ in the brain. The optically monitored parameters were compared with conventional NICU monitors and Xe-CT. The LDF signals were low with median and 25 to 75% interquartiles of perfusion = 70 (59 to 83) a.u. and TLI = 2.0 (1.0 to 2.4) a.u. and showed correlation with the NICU monitors in terms of heart rate. Median and interquartiles of SO_2_ were 17.4 (15.7 to 19.8) %. The lack of correlation between local perfusion and cerebral perfusion pressure indicated intact cerebral autoregulation. The systems were capable of monitoring both local perfusion and SO_2_ with stable signals in the NICU over 4 days. Further clinical studies are required to evaluate the optical systems’ potential for assessing the onset of secondary brain injury.

## Introduction

Monitoring of local brain microcirculation and oxygenation saturation (SO_2_) is a clinical demand not yet fully addressed in neurointensive care. Cerebral blood flow (CBF) monitoring is especially important for patient groups treated for traumatic brain injury (TBI) or subarachnoid hemorrhage (SAH) due to the increased risk of secondary insults coupled with high mortality [17, 38]. To detect the onset of delayed cerebral ischemia, there is a need for sensitive and continuous monitoring devices to assess brain function in terms of local blood flow and oxygenation [20].

The commercially available brain monitors use invasive catheters to register intracranial pressure (ICP), cerebral perfusion pressure (CPP), and metabolic biochemistry using microdialysis (MD) [8]. The vulnerability to local ischemia in patients is often detected at a late stage, sometimes in contradiction with ICP readings within the normal healthy range [27]. Oxygen microelectrodes such as the Licox® system are sometimes used for local oxygen assessment in the brain but have a relatively slow response time to changes [26]. The electrodes measure cerebral oxygen pressure (pO_2_) related to SO_2_ through the oxygen dissociation curve (ODC). Techniques such as jugular venous SO_2_ and blood flow monitoring give a general view of the systemic parameters of the brain but do not reflect regional impairments such as focal ischemia [33]. Local cerebral blood flow can be indirectly estimated using thermal gradients by means of thermodilution (TD, e.g., the Hemedex® system), but problems with baseline drift have been reported [12]. Transcranial Doppler ultrasound (TCD) is a noninvasive technique that can be used to assess CBF by measuring the blood flow velocity typically in the middle cerebral artery and detect changes globally [1]. Current imaging techniques which assess metabolic information of the brain such as xenon-enhanced computed tomography (Xe-CT) [7], positron emission tomography, or functional magnetic resonance imaging provide only “snapshot” information and are used intermittently with long time intervals in between measurements [25]. Moreover, these imaging techniques are relatively expensive and some expose patients to ionizing radiation.

Optical techniques have the advantage of providing a real-time response and continuous monitoring with high temporal resolution during longer periods. Different optical modalities can also be combined into one probe for parallel monitoring of multiple parameters. Examples are laser Doppler flowmetry (LDF) for estimation of microvascular blood flow also denoted perfusion [23, 24] and diffuse reflectance spectroscopy (DRS) [2, 14] for estimating blood SO_2_. DRS can be analyzed to derive information of chromophore content including hemoglobin as it uses a broad range of wavelengths in the visible spectrum. In this study, DRS was preferred over near-infrared spectroscopy (NIRS) in terms of its localized sampling volume through small optical fiber separations in the sensing probe and use of a wide wavelength range in the visible spectrum compared to only using a few wavelengths in the NIR region [6]. Examples of work for assessing human brain circulation with optics are Martini et al. and Klein et al. using white light spectroscopy and NIRS [18, 21] or recent work using DRS [29, 30].

The LDF technique has been extensively applied in the field of dermatology [34]. LDF has also been used as a tool in various research applications. Some examples are animal models of stroke [4] and brain tissue lesioning [40]. Other examples are evaluation of myocardial perfusion during heart surgery [16] and assessment of human brain microcirculation in relation to SAH [3]. LDF has also been used for recordings along stereotactic trajectories during deep brain stimulation implantations where the total backscattered light intensity (TLI), apart from the perfusion signal, can be used to relate to the anatomical position [42, 43]. Rejmstad et al. recently used LDF [31] and DRS [30] to investigate the cerebral microcirculation. In this application, a thin flexible probe was used during open brain tumor surgery. These intraoperative recordings showed that a stable LDF signal could be achieved during at least 15 min. In the present study, the optical probe was used for long-term monitoring of microvascular perfusion and SO_2_. The aim of this study was to evaluate LDF and DRS for monitoring of brain microcirculation in the neurointensive care unit (NICU). The methodology is exemplified in monitoring of a patient over 4 days. The physiological parameters recorded with the optical systems were compared with values from the standard monitors in the NICU.

## Material and methods

### Optical monitoring systems

**Fig. 1:**
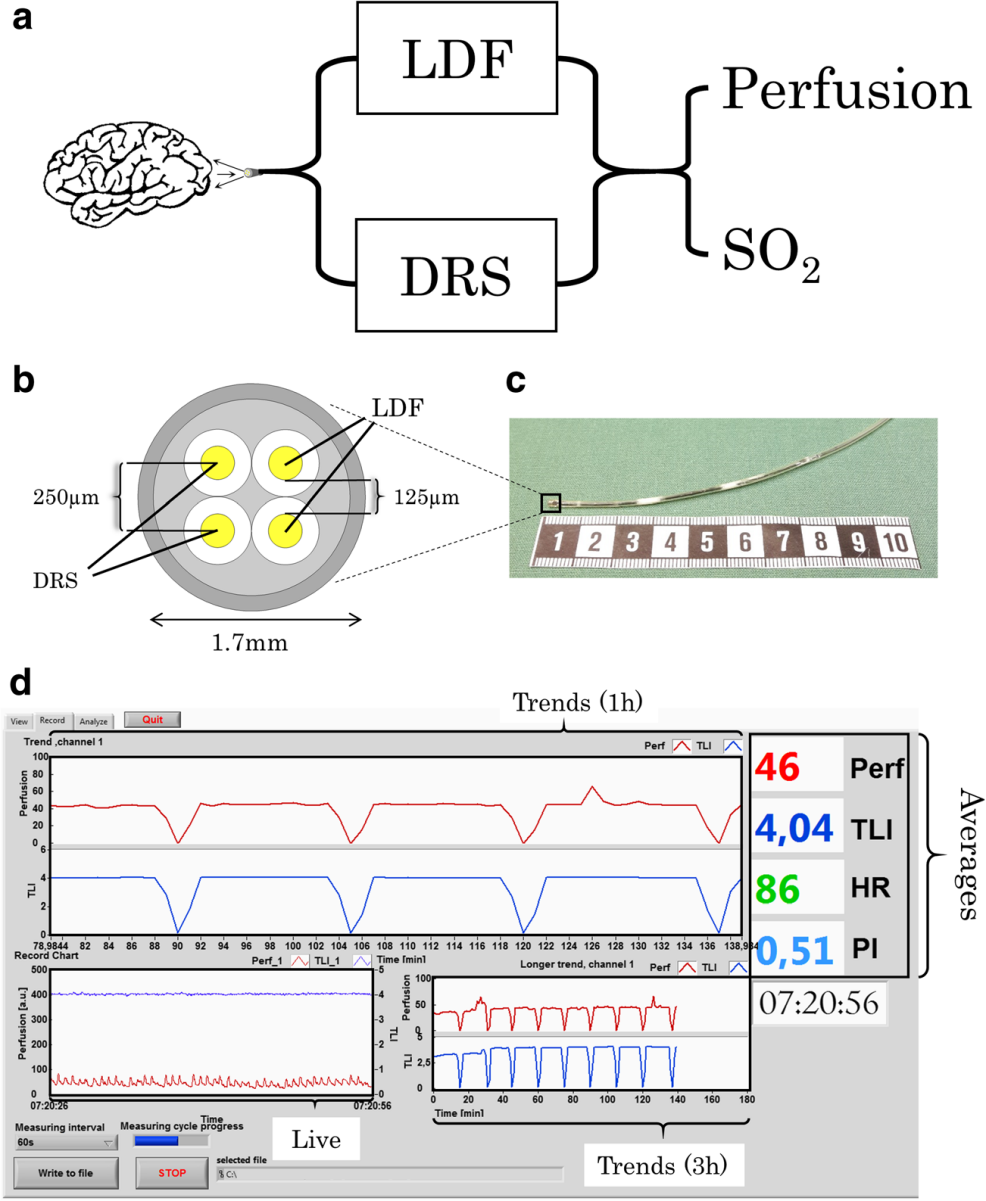
**a** System overview with laser Doppler flowmetry (LDF) and diffuse reflectance spectroscopy (DRS) to monitor microcirculatory perfusion and oxygen saturation, **b** probe tip configuration with one pair of optical fibers for diffuse reflectance spectroscopy (DRS) and one pair for laser Doppler flowmetry (LDF), **c** photo of the probe tip next to a 10-cm reference scale, and **d** user interface for the LDF software displaying parameter averages, live signals, and signal trends where drops in the trends correspond to interruption in the LDF signals during DRS recordings

An overview of the optical systems for monitoring perfusion and SO_2_ is displayed in Fig. 1a. The systems comprised of an LDF device (Periflux 5000, Perimed AB, Sweden), a DRS setup using a spectrometer (AvaSpec 2048-2, Avantes, the Netherlands), a light source in the visible wavelength range (AvaLight HAL-S, Avantes), and a laptop. The ranges of the signals from the LDF system were 0–999 arbitrary units (a.u.) for perfusion and 0–10 a.u. for the TLI. All components were positioned on a portable trolley and the systems were fit for a confined setting such as bedside in an NICU. The LDF system was calibrated using a motility standard (Perimed AB, Sweden) with particles that exhibit Brownian motion and the DRS system to a white tile reference.

A custom-made optical probe (Fig. 1b, c) was designed and built in-house for the purpose of monitoring parameters in the NICU environment. The probe’s design had previously been evaluated in short-term measurements during brain tumor resection [31]. To enable smooth insertion and easy fixation similar to that of a ventricular drain, or ICP and microdialysis catheters, the probe had a catheter-like design and a thin flexible tip (*Ø* = 1.7 mm). In the core of the probe, four optical fibers (*Ø*_core_ = 125 μm, numerical aperture = 0.37) were placed adjacent to each other with a center-center distance of approximately 250 μm. Each fiber pair was assigned to sending and receiving light to one of the two systems of LDF and DRS. In the center of the probe, a 6-cm-long thin stainless steel wire was placed in order to ascertain visibility of the probe placement during X-ray examination. A 5-m-long probe cable between the LDF-DRS equipment and the probe tip was used so the measurement equipment could be placed bedside at the patient’s foot end. The probe was sterilized before the measurements using a radiation-based low temperature procedure referred to as Sterrad® [11].

Software modules were in-house programmed in LabVIEW® (National Instruments Inc., USA) for recording and real-time display of signals generated by each system in the NICU [29, 31]. The LDF software module contains features such as trend monitoring of the perfusion and TLI signals by averaging over a preselected time interval (e.g., 10, 20, 30, 60 s) and calculation of the heart rate (HR) as well as the pulsativity index (PI) (Fig. 1d). The HR was estimated using a systolic peak finding algorithm with the LDF-generated perfusion signal and PI as the amplitude difference of the flow pulsations divided by the average signal over the selected interval [31]. The SO_2_ in the tissue was estimated using an algorithm previously developed for use in human cerebral white matter [29] and evaluated using clinical data [28].

### Surgical procedure and probe placement

The patient in this study suffered from an SAH due to a ruptured aneurysm of the left middle cerebral artery. The patient was unconscious when admitted at the local hospital (6 in the Glasgow Coma Scale) and was acutely referred to the Neurosurgical Department at Linköping University Hospital. Before enrolment of the patient in the study, informed written consent was given by the patient’s immediate family. The study was approved by the local ethics committee (No. M182-04, 2010/359-32).

**Fig. 2:**
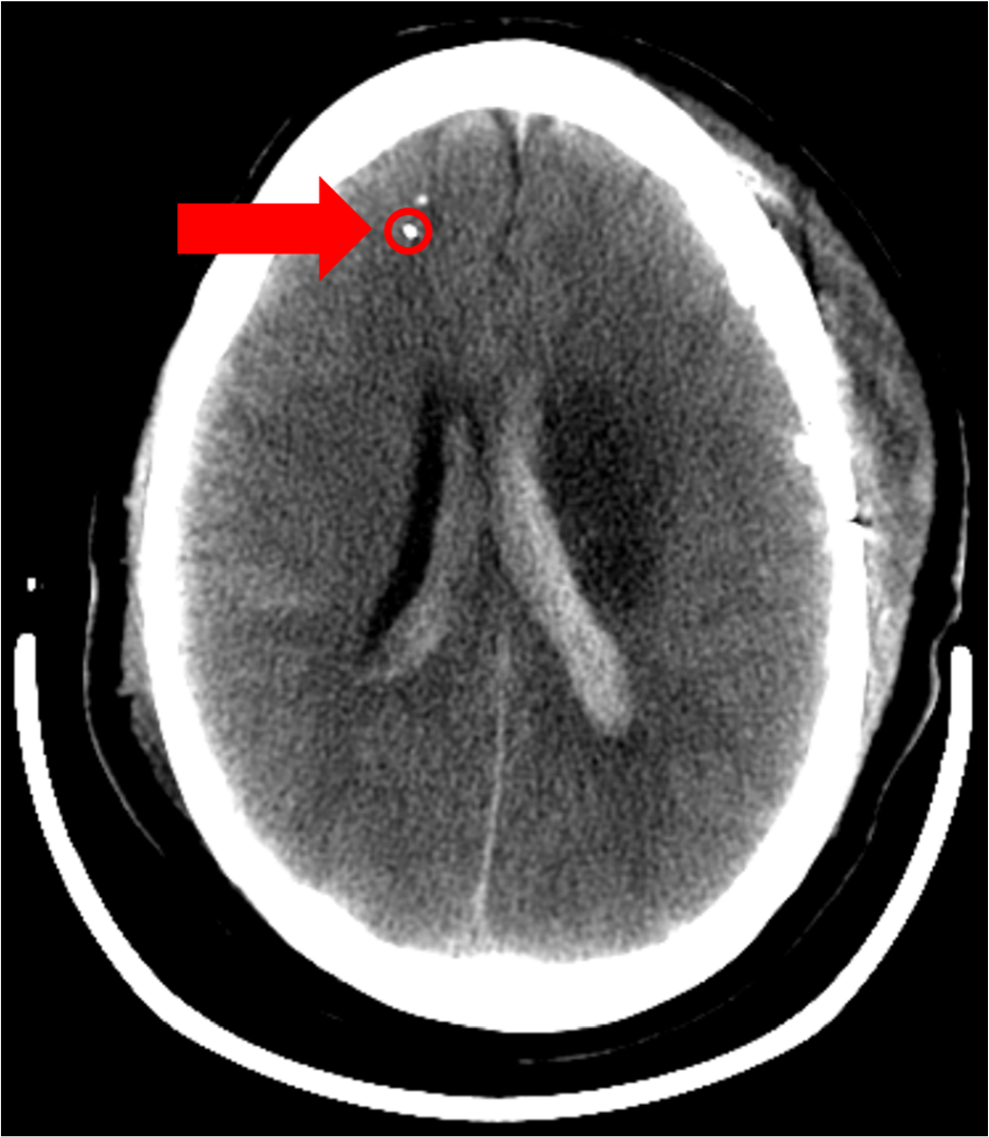
Probe placement with a CT scan from the patient where the red circle marks the optical probe positioned next to a microdialysis catheter

A pair of microdialysis catheters was placed bilateral into the patient’s brain during a routine surgical procedure in which the optical probe was placed parallel next to one microdialysis catheter. The optical probe was manually inserted 2–3 cm into the white brain matter (intraparenchymal) in the water shed area of the right frontal lobe as seen in the CT image in Fig. 2. The probe’s position was secured by a fixation wing on the skin surface next to the entry of the probe. The optical probe was equivalent with external ventricular drains and ICP monitors in terms of the securing and implantation procedure. After probe fixation in the operating room, initial recordings by LDF and DRS were made to assert the signal quality before the patient was transferred to the NICU. The systems were disconnected from the probe before transferring the patient to the NICU.

### Neurointensive care measurements

**Fig. 3:**
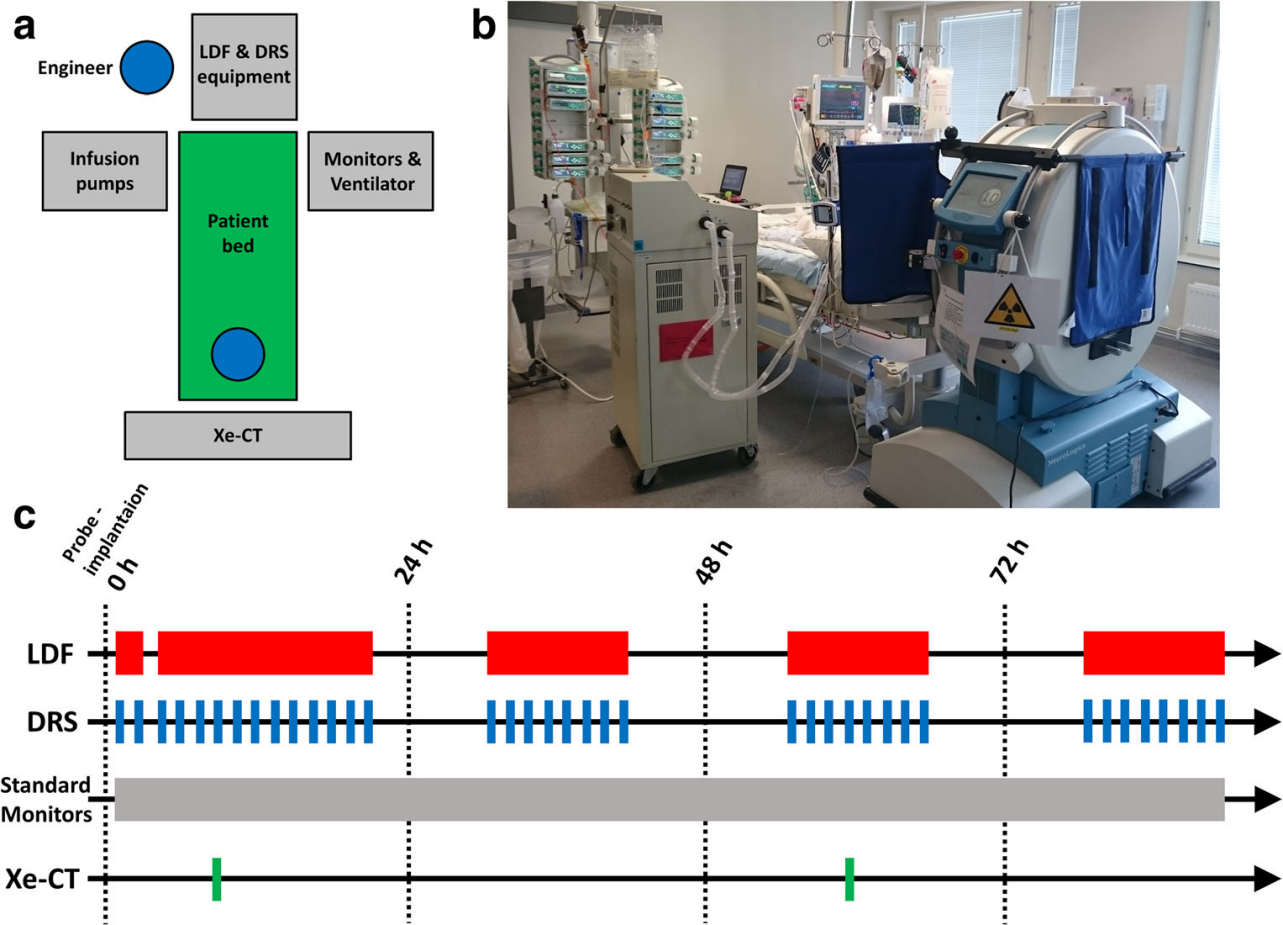
**a** Positioning of technical equipment in relation to the patient in the neurointensive care unit, **b** photo displaying the clinical setting with a mobile Xe-CT scanner, and **c** 96-h measurement timeline where approximate occasions of data acquisition using different modalities are marked

After arrival to the NICU, the probe was connected to the optical system again. Figure 3a, b shows the positioning of the equipment in relation to the patient where the optical systems were placed at the foot end. The optical measurements resumed and performed in parallel to the patient’s treatment and standard monitoring in the NICU at Linköping University Hospital (Fig. 3c). The LDF signals (perfusion and TLI) were acquired for the first 15 h after surgery and later for approximately 10 h during daytime on the following 3 days (around 40 h in total). The perfusion and TLI were continuously displayed in real-time or as trends during the measurements together with estimated HR and PI. The LDF signals were sampled with 100 Hz and a time constant (*τ*) of 0.03 on the Periflux device. The software was set to display average LDF parameters and trends using 60-s intervals. Tissue SO_2_ was calculated and presented in real time from gathered DRS spectra every 15 min for the first day, and every 30 to 60 min during the following 3 days (*n* = 70). The SO_2_ was estimated in real time every 15 to 60 min where each occasion lasted 1–2 min with three separate 30 s collections of spectra using an integration time of 10 ms. During each DRS measurement (approximately 1–2 min), the probe contact to the LDF laser was disconnected in order not to influence the DRS signal. An engineer present during the entire measurement procedure took notes of different treatment and measurement-related events. Specific patient treatments, such as bandage change, mouth care, and drug administration, were noted. This was also made in order to keep track of external movements that may produce artifacts in the LDF measurement. Example of such events is when the head of the patient bed was tilted from a horizontal to a sitting position or when treatments such as mouth care and bandage change were performed.

Data from the standard monitoring equipment in the NICU (IntelliVue MP70 patient monitor, Philips, USA) was collected. Data logs with 15-min averages of parameters such as HR from the electrocardiogram (ECG), ICP, CPP, and SO_2_ from pulse oximetry were extracted from the monitors. According to the clinics’ routine, a mobile CT scanner (CereTom, NeuroLogica Corp., USA) was used together with xenon gas to map the cerebral blood flow (Fig. 3b). An overview of the measurement timeline can be found in Fig. 3c.

### Data analysis

The parameters derived from LDF signals (perfusion, TLI, HR, and PI) were analyzed by averaging over 15 min. LDF signals affected by movement artifacts and lack of signal when the LDF laser was disconnected during DRS measurements were removed from the analysis using a fixed threshold. From each time set of local SO_2_ recordings, the three spectra were averaged.

Correlation between the 15-min averages for the perfusion, SO_2_, ICP, and CPP parameters were tested with Pearson’s correlation coefficient using Matlab® 2015 (MathWorks Inc., USA). The median and values of the first and third interquartiles (Q1 to Q3) were used as the data were not normally distributed as investigated by Anderson-Darling tests (*p* < 0.05) using Minitab® (Minitab Inc., UK). Statistical tests (Kruskal-Wallis method) were performed to investigate the difference among the perfusion, SO_2_, ICP, and CCP data sets between each day of monitoring. A *p* < 0.05 was considered significant.

## Results

**Fig. 4:**
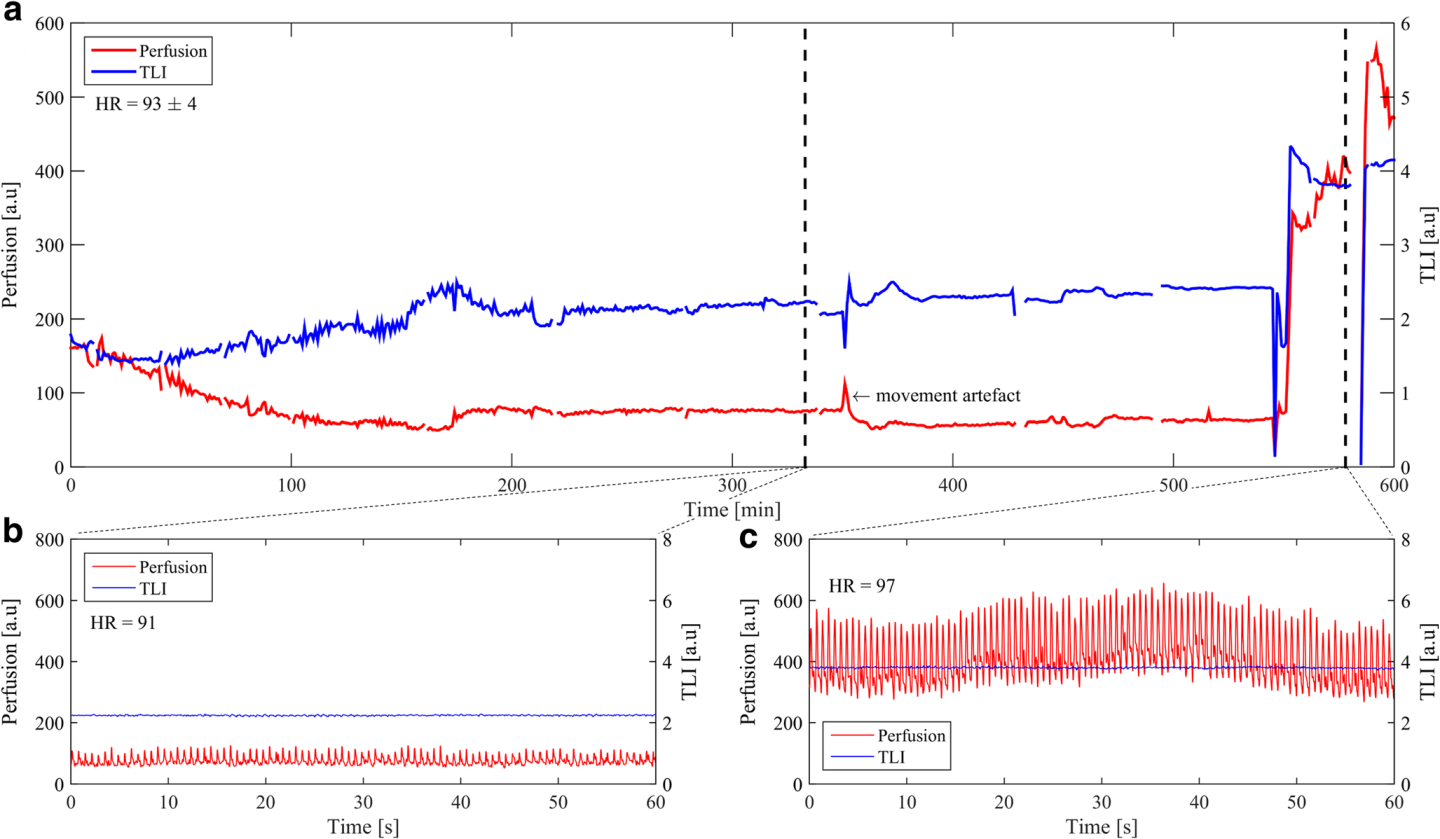
**a** Laser Doppler flowmetry (LDF) signal with 1-min averages over a 10-h period of day 2 where the two dashed lines represent the sections for part b and c in the current figure, **b** 1-min section of the LDF signal with low perfusion, and **c** 1-min section of the LDF signal with higher perfusion

**Fig. 5:**
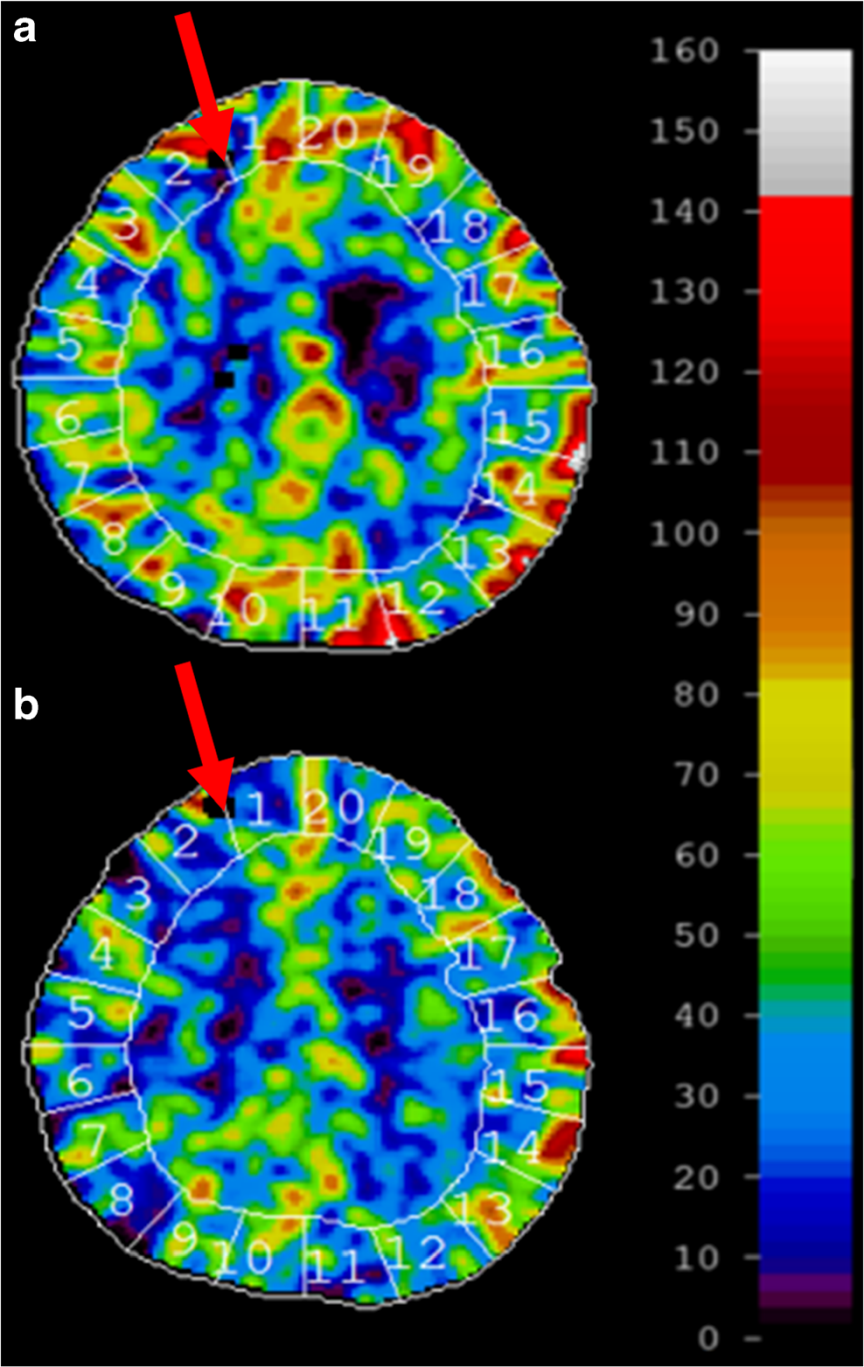
Scans from a Xe-CT (**a**) day 1 and (**b**) day 3. The color bar shows blood flow values in ml/100 g/min; the arrows point at the position of the optical probe tip. The blood flow in the region neighboring the optical probe was approximately 11 and 27 ml/100 g/min at day 1 and day 3, respectively

The optical signals were stable during the 4 days of monitoring. A summary of the median values is presented in Table 1. No indication of blood clots or tissue encapsulation disturbed the signal quality. The optical probe was easily retracted in the NICU after the optical monitoring. Real-time signals were displayed next to the NICU during the entire LDF measurement procedure. Typical perfusion and TLI trend curves from day 2 over a time period of 10 h with 60-s averaging are presented in Fig. 4a with close-ups presenting data over 1-min intervals in Fig. 4b, c showing low and higher perfusion variations. The perfusion signal increased after repositioning of the patient (Fig. 4a at approximately 560 min). Xenon-enhanced CT images collected days 1 and 3 after the probe placement are presented in Fig. 5. The perfusion for the region close to the optical probe measured with the Xe-CT resulted in values of approximately 11 ml/100 g/min and 27 ml/100 g/min 1 and 3 days after probe placement. The corresponding LDF values were 52 and 72 a.u., respectively.

**Table 1:** Calculated median values and quantiles (Q1 to Q3) from the 15-min averages during each day of monitoring for perfusion (Perf), total light intensity (TLI), intra cranial pressure (ICP), cerebral perfusion pressure (CPP), and oxygen saturation (SO_2_)

	Day 1	Day 2	Day 3	Day 4	Days 1 to 4	*n*
Perf [a.u.]	57 (43–67)	75 (61–82)	74 (70–85)	76 (68–118)	70 (59–83)	157
TLI [a.u.]	2.8 (2.3–3.9)	2.2 (1.9–2.4)	0.7 (0.5–0.9)	1.0 (0.9–1.2)	2.0 (1.0–2.4)	157
ICP [mmHg]	19 (17–20)	15 (14–18)	13 (13–15)	10 (10–11)	15 (13–19)	155
CPP [mmHg]	66 (62–70)	70 (62–73)	77 (73–81)	99 (88–103)	71 (64–79)	155
SO_2_ [%]	17.0 (15.5–19.9)	17.4 (16.3–21.0)	18.9 (17.8–19.4)	17.1 (16.7–17.3)	17.4 (15.7–19.8)	70

**Fig. 6:**
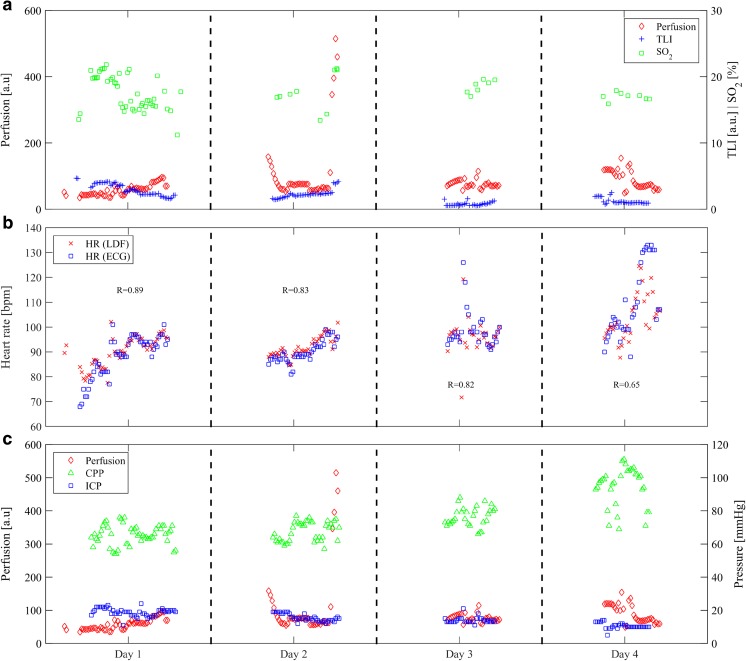
**a** Perfusion and TLI from laser Doppler flowmetry (LDF) and SO_2_ from diffuse reflectance spectroscopy collected over 4 days. **b** Heart rate estimation based on the LDF signal and values from the standard electrocardiogram (ECG) monitor. **c** Comparison between perfusion, cerebral perfusion pressure (CPP), and intracranial pressure (ICP)

**Fig. 7:**
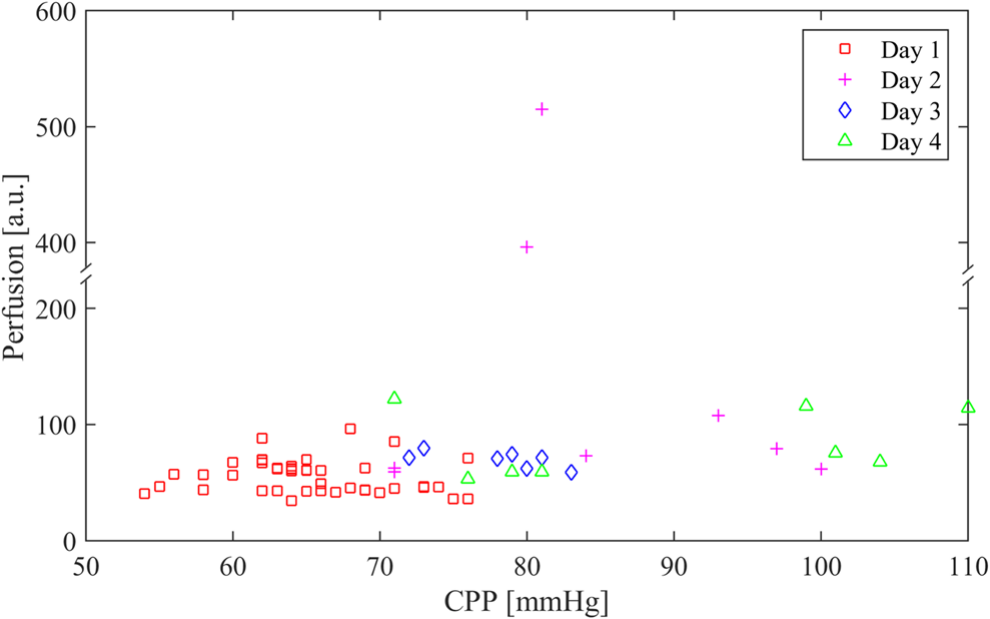
Perfusion over 4 days plotted against the cerebral perfusion pressure in analogy with the conventional Lassen’s curve

Figure 6a shows 15-min averages of the perfusion, TLI, and SO_2_. The medians for all 4 days were 70 (59 to 83) a.u. for the perfusion and 2.0 (1.0 to 2.4) a.u. for the TLI, as seen in Table 1. The estimated intraparenchymal SO_2_ over 4 days showed a median of 17.4 (15.7 to 19.8) %. The HR estimation from the LDF signal showed correlation (*r* = 0.86, *n* = 155) with the HR from the standard ECG monitor, Fig. 6b. No significant difference was found between different days. The ICP, Fig. 6c, had a median of 15 (13 to 19) mmHg over 4 days and was highest on the first day of monitoring (19 mmHg) compared with the following days where it decreased to 10 mmHg (Table 1). The median CPP, Fig. 6c, was 71 (64 to 79) mmHg and was lower on the first 2 days, 66 mmHg, compared with the following days where it increased up to 98 mmHg. The flat geometrical shape of the relation between perfusion and CPP in Fig. 7 and the low correlation coefficients between the two parameters indicate intact autoregulation according to the principle of the Lassen’s curve.

## Discussion

After 4 days and 40 h of optical monitoring in the NICU using LDF and DRS, the signal quality remained unchanged where it was still possible to record variations in local blood flow and oxygenation. The LDF and DRS systems have a potential to supplement the standard monitors in the NICU and provide information regarding cerebral autoregulation and local circulation that can help to detect focal ischemia in areas at risk.

### Practical aspects

The catheter-like probe was tightly secured to the patient in a similar way to the standard ICP monitors or external ventricular drains. The LDF signals detected variations in the perfusion and TLI, Fig. 5, possibly from small movements of the probe tip as a result of the patient treatment or of reduced brain swelling indicated by the decrease in ICP (Table 1). Apart from occasional movement artifacts, the optical signals were stable over 4 days without any significant reduction in signal quality, i.e., perfusion and TLI that could have indicated potential fibrous capsulation or blood clotting [43]. For applications susceptible to motion, the motion artifacts could potentially be detected and tracked using signal analysis described by Humeau et al. [10].

An advantage of using the LDF method is that it measures the tissue’s microvascular blood flow, i.e., the average velocity multiplied by the concentration of moving red blood cells in the apposed tissue volume [23] compared with indirect methods such as clearance or dilution techniques (e.g., TD) [20]. The small size offered by using fiber optic probes is essential in endoscopic and catheterization applications such as in the current NICU setting. The use of optical techniques enables multimodal exploration using a single probe setup that helps to minimize the risk of infection associated with introducing multiple objects into the brain.

### Monitored parameters

Detecting the onset of secondary brain injury through indications of events such as vasospasm or impaired microcirculation in vulnerable areas of the brain is not yet standardized in the NICU making multimodal optical monitoring a potential tool to evaluate the patient status and to predict tendencies of deterioration. Assessing ischemia using imaging methods such as PET limits the analysis to stable patients and captures a point in time whereas the dynamic process of local ischemia may occur during short periods that may remain undetected without continuous monitoring [33]. This is one of the main reasons for suggesting optical systems of LDF and DRS as complements to existing monitoring methods providing continuous real-time recordings of microcirculatory parameters.

The high spatial variability of microvascular blood flow has a large effect on the resulting signal acquired with LDF when using a probe-based system. As an example, the perfusion and TLI produced by LDF display an inverse relation when measuring in brain tissue due to a higher degree of vascularization and blood flow in gray matter compared to white matter as shown by earlier studies [42, 43]. This effect can be an explanation to the appearance of the LDF signal in the first 180 min of Fig. 4a, where the reduced brain swelling could have caused the probe to a slight transition from a position in mainly gray brain matter into a locally more white tissue region which complies with the inverse relation between the two parameters of perfusion and TLI. The sampled regions in the Xe-CT measurements are much larger compared with the local sampling of the LDF probe with a forward looking distance of about 0.5 to 1.5 mm in brain tissue [5, 13]. It is also seen in the Xe-CT images that the blood flow variability is large between different spatial points. The presence of a thin metal wire in the tip of the optical probe produces artifacts in the CT images. This, however, limits a precise comparison in terms of CBF between LDF and Xe-CT where values next to the probe artifact were extracted in the Xe-CT image for comparison. This artifact together with spatial blood flow variation in the brain makes it difficult to directly compare the blood flow from the Xe-CT images in the vicinity of the optical probe (Fig. 5) with the recorded LDF perfusion values. Similar spatial variations are also found for other tissues, e.g., skin [41]. The advantage of the LDF monitoring method is its temporal component and possibility to follow changes of the microvascular blood flow over time [39]. The heart rate estimation from the LDF signal gave a correlation of 0.86, Fig. 6, compared with the standard ECG monitor. This correlation value would undoubtedly increase if the algorithm was to be used together with additional signal filtering and fine-tuned in order to remove influence of signal variations such as from autoregulatory slow-changing waves and motion artifacts.

The SO_2_ estimation from the DRS signals represents an average in the sampled tissue volume where the microvascular blood found in arterioles and venules contributes to the signal. The relatively low values of SO_2_ measured with DRS should not to be confused with arterial SO_2_ from blood gas analysis or pulse oximetry readings. Similar SO_2_ level was found in white matter during DRS measurements in relation to DBS implantation surgery [28, 30]. The baseline values of cerebral pO_2_, pCO_2_, pH, and temperature reported by Hoffman et al. [9] (20 mmHg, 50 mmHg, 7.15 and 37 °C) correspond to an SO_2_ value of 30.8% according to the ODC of hemoglobin described by Severinghaus [36]. The range of normal intraparenchymal pO_2_ from 20 to 35 mmHg [32] results in SO_2_ values of approximately 30–60% using the ODC for normal physiological conditions. These values are somewhat higher than those estimated for white matter using the described method [30]. However, in this application, tracking physiological changes over time is more important than the absolute accuracy of individual measurements. Sommer et al. recently measured SO_2_ using spectroscopy in human brain tissue in 20 SAH patients where the mean SO_2_ was around 39% at a tissue depth of 7 mm, which is comparable to estimations in a previous study with recordings from more than 150 well-defined sites in relation to stereotactic deep brain stimulation implantations using the same algorithm as was used here [30, 37]. Another study with a similar spectroscopic approach describes an estimated SO_2_ of 26% in white brain matter at a tissue depth of 33 mm [15].

The relation between the perfusion and the CPP was plotted in Fig. 7 similar to CBF and CPP in Lassen’s curve where the geometrical shape represents the autoregulation. Lassen’s curve is a plot of CBF against CPP that indicates intact cerebral autoregulation through a constant blood flow over variations in CPP at the plateau that normally resides between 60 and 150 mmHg in CPP [22]. A linear relation in Lassen’s curve indicates impaired autoregulation or ischemic conditions. The correlation between perfusion measured by LDF and CPP could possibly also, in accordance with Lassen’s curve, be used to predict deterioration or improved autoregulation according to Lam et al. [19]. A similar approach using the correlation between SO_2_ and CPP has been suggested by Sekhon et al. [35] where a correlation coefficient higher than 0.3 indicates impaired cerebral autoregulation. The flat shape of the relation between perfusion and CPP in Fig. 7 indicated an overall intact autoregulation during the 4 days of monitoring with exception for the two outliers at day 2 related to the increased perfusion after repositioning of the patient. The relation between the measured perfusion or SO_2_ and the CPP produced low correlation coefficient values. Absence of correlation between perfusion or SO_2_ and CPP represents intact autoregulation in accordance with Lam et al. and Sekhon et al. [19, 35]. An advantage of using the suggested monitoring system is its potential to directly assess the status of the cerebral autoregulation through the perfusion and SO_2_ parameters. However, more measurements are needed to fully prove the concept.

### Future work

In this study, the DRS was used intermittently every 15 to 60 min while disconnecting the laser source of the LDF system; therefore, a future improvement could be to equip the system with an optical filter that would diminish the laser light before reaching the spectroscope in a way that the LDF and DRS systems could be used simultaneously. This would enable continuous recording of spectral information and oxygen estimation in parallel with the perfusion measurement.

The system offers the capability of extending the number of optical probes to enable bilateral monitoring for assessing differences in physiological parameters close to the damaged tissue and contralateral in unaffected tissue. Investigation of optimal probe placement is another concern in terms of vasospasm detection and assessment of autoregulation.

Yokose et al. [44] proposed in 2010 that time-resolved NIRS may in the future be used to detect delayed vasospasm after SAH based on the results in 14 SAH patients and 11 healthy controls that showed reduced SO_2_ and hemoglobin concentration during vasospasm. A comparison between the LDF-DRS setup and other local brain monitoring systems such as Hemedex® thermodilution, Licox® pO_2_-electrodes, and microdialysis would further help to reveal advantages and provide insight of the monitoring capability of the suggested optical system. Furthermore, measurements on more patients are needed to define thresholds for ischemia, hypoxia, and vasospasm compared to a healthy status.

## Conclusion

The LDF and DRS systems were evaluated on one neurointensive care patient for a period of 4 days with stable signals. The obtained parameters perfusion and SO_2_ showed to have a potential for investigating cerebral autoregulation. Further studies are needed to investigate and validate the optical systems’ potential for assessing the onset of secondary brain injury, since no indication of such events was present in the standard monitoring equipment during these measurements. This initial study clearly showed that the suggested combination of systems enabled monitoring of both local perfusion and SO_2_ using a catheter-like fiber optic probe in the neurointensive care unit.
